# Analytical performance of the digital morphology analyzer Sysmex DI-60 for body fluid cell differential counts

**DOI:** 10.1371/journal.pone.0288551

**Published:** 2023-07-27

**Authors:** Sumi Yoon, Hye Ryoun Kim

**Affiliations:** Department of Laboratory Medicine, Chung-Ang University College of Medicine, Seoul, Republic of Korea; The Ohio State University, UNITED STATES

## Abstract

**Background:**

Sysmex DI-60 (Sysmex, Kobe, Japan) is a digital morphology (DM) analyzer widely used in clinical laboratories and supports body fluid (BF) applications. We evaluated analytical performance of DI-60 compared with XN-350 (Sysmex) and manual counting for BF cell differential counts.

**Methods:**

A total of 213 BF samples were collected (47 cerebrospinal fluid [CSF], 80 pleural fluid, and 86 ascites samples). The analytical performance of DI-60 for BF cell differential counts was evaluated based on sensitivity, specificity, accuracy, and agreement. BF cell differential counts obtained by DI-60 were compared with those obtained by XN-350 and manual counting.

**Results:**

The overall sensitivity was high for neutrophils, lymphocytes, and macrophages (range, 83.1–99.4%). The overall specificity and overall accuracy were high for all cell types (range, 95.3–99.7% and 94.3–99.3%, respectively). The agreement between DI-60 pre-classification and verification was strong (κ = 0.89). The absolute mean differences between DI-60 verification and XN-350 ranged from 0.26 to 11.05, and differences between DI-60 verification and manual counting ranged from 0.01 to 4.76.

**Conclusions:**

This is the first study to evaluate the performance of DI-60 compared with XN-350 and manual counting for BF cell differential counts. DI-60 showed reliable performance with CSF, pleural fluid, and ascites samples. For BF cell differential counts, DI-60 may be a better option than XN-350 and could be used for screening purposes in understaffed laboratories. To improve the hematology workflow for BF cell differential counting, the DM analyzer needs to be optimized by taking into account the laboratory situation and unmet needs, and the clinical laboratory needs to establish criteria for verification and manual slide review.

## Introduction

Cytological examination and cell differential counting of body fluid (BF) samples are required for diagnosing and monitoring various diseases, including hematologic disease, and determining treatment plans [1]. White blood cell (WBC) counts are increased in cerebrospinal fluid (CSF) samples in neuroinflammatory diseases such as meningitis and encephalitis, and the potential cause (e.g., bacterial or viral) can be identified by cell differential counting [1, 2]. The detection of malignant cells in serous BF samples such as pleural fluid and ascites is important for disease staging and treatment [3]. A manual method using a light microscope is still the gold standard for BF cell enumeration and differential counting, which is a combination of quantitative assessment using a hemocytometer chamber and morphological assessment using a Romanowsky-stained slide of cytocentrifuged BF [4]. However, this approach is time/labor-intensive, complex, and subjective with high intra/inter-observer variability and requires experienced and trained personnel [1–3, 5, 6].

With technological advancement, automated hematology analyzers and digital morphology (DM) analyzers have been widely used for cell enumeration and differential counting of BF and peripheral blood (PB) samples [7–10]. The automated hematology analyzers include Sysmex XN and XN-L series (Sysmex, Kobe, Japan), BC-6800 (Mindray, Shenzhen, China), and Unicel DxH series (Beckman Coulter, Brea, CA, USA) and the DM analyzers include CellaVision DM96 (DM96; CellaVision AB, Lund, Sweden) and Sysmex DI-60 (DI-60; Sysmex) [7–10]. Their use is expected to reduce turnaround time and inter-observer variability and improve precision [5, 7, 11]. In addition, DM analyzers can reduce eyestrain caused by microscopy and facilitate morphological education and discussion as the results can be digitally archived and accessed remotely [7, 12].

According to the International Council for Standardization in Hematology (ICSH) guidelines, verification of an automated analyzer should be performed by each laboratory before it is used for routine testing [13]. Verification of an automated analyzer for BF cell enumeration should not be considered different from verification of an automated analyzer for cell enumeration of PB samples [13]. Various studies have been conducted to evaluate the performance of automated hematology analyzers compared with manual counting using BF samples [1–3, 5, 6, 10, 11, 14, 15]. However, only a few studies have evaluated the performance of DM analyzers using BF samples [7–9]. In particular, only one study compared DI-60 with manual counting for BF cell differential counts [8]. To the best of our knowledge, no study has comprehensively evaluated the analytical performance of DI-60 for BF cell differential counts. Although both the DM analyzer and the automated hematology analyzer perform cell differential counting in BF samples, no study has compared these two analyzers with different principles of cell differential counting. In this study, we aimed to evaluate the analytical performance of DI-60 for BF cell differential counts using CSF, pleural fluid, and ascites samples. We also compared BF cell differential counts obtained by DI-60 with those obtained by the automated hematology analyzer Sysmex XN-350 (XN-350; Sysmex) and manual counting in order to identify which analyzer is more recommended for BF cell differential counting.

## Materials and methods

### Study samples

This study was conducted at Chung-Ang University Hospital (CAUH), Seoul, Republic of Korea, from July to December 2022 using the results of BF cell differential counts in medical records and archived samples. A total of 213 BF samples (47 CSF, 80 pleural fluid, and 86 ascites samples) and their medical records were collected (**Table 1**). These BF samples were obtained from subjects (median age, 66 years; interquartile range [IQR], 49–78 years) whose BF cell differential counts were requested for diagnosing diseases or monitoring health conditions from May to August 2022. The samples were collected in BD Vacutainer^®^ K2 EDTA tubes (BD, Franklin Lakes, NJ, USA) and analyzed by XN-350 within 2 h after collection. Cytospin slides were prepared within 2 h after collection using the Shandon Cytospin 4 Cytocentrifuge (Thermo Fisher Scientific, Waltham, MA, USA) at 1,000 rpm for 4 min and stained using SP-50 (Sysmex) with Wright-Giemsa (RAL Diagnostics, Martillac, France).

**Table 1:** Sample characteristics.

	Total (n = 213)	CSF (n = 47)	Pleural fluid (n = 80)	Ascites (n = 86)
Age, median (IQR)	66 (49–78)	61 (29–74)	78 (69–82)	54 (45–71)
Male, n (5)	134 (62.9)	25 (53.2)	50 (62.5)	59 (68.6)
Diagnosis, n (%)				
Infectious/inflammatory diseases^†^	51 (23.9)	16 (34.0)	29 (36.2)	6 (7.0)
Hematologic malignancies^‡^	17 (8.0)	9 (19.1)	7 (8.8)	1 (1.2)
Non-hematologic malignancies^§^	42 (19.7)	1 (2.1)	23 (28.7)	18 (20.9)
Alcoholic liver cirrhosis	51 (23.9)	-	2 (2.5)	49 (57.0)
Other diseases^¶^	52 (24.5)	21 (44.7)	19 (23.7)	12 (14.0)
WBC counts, median (IQR)	198 (73–565)	33 (3–140)	510 (199–1,787)	168 (89–284)

^†^Infectious/inflammatory diseases include pneumonia (n = 22), meningitis/meningoencephalitis (n = 14), spontaneous bacterial peritonitis (n = 5), pericarditis (n = 3), pleusy (n = 3), cholecystitis (n = 1), hepatitis A (n = 1), herpangina (n = 1), and neuromyelitis optica (n = 1).

^‡^Hematologic malignancies include malignant lymphoma (n = 9), myelodysplastic neoplasms (n = 3), chronic myeloid leukemia (n = 2), Waldenström macroglobulinemia (n = 2), and acute lymphoblastic leukemia (n = 1).

^§^Non-hematologic malignancy includes pancreatic cancer (n = 13), lung cancer (n = 12), hepatocellular carcinoma (n = 5), breast cancer (n = 4), cholangiocarcinoma/gallbladder cancer (n = 2), mesothelioma (n = 2), esophageal cancer (n = 1), glioblastoma (n = 1), kidney cancer (n = 1), and rectal cancer (n = 1).

^¶^Other diseases include cardiovascular diseases (n = 9), cerebral hemorrhage (n = 9), non-alcoholic liver cirrhosis (n = 8), pneumothorax/hemothorax/chylothorax (n = 6), end stage renal disease (n = 4), cerebral infarction (n = 3), delirium (n = 3), hydrocephalus (n = 2), acute respiratory failure (n = 1), acute liver failure (n = 1), azotemia (n = 1), brain epidermoid cyst (n = 1), cerebral aneurysm (n = 1), diaphragm eventration (n = 1), jaundice of unknown origin (n = 1), and seizure (n = 1).

Abbreviations: CSF, cerebrospinal fluid; IQR, interquartile range; WBC, white blood cell.

### BF cell differential counts obtained by DI-60, XN-350, and manual counting

DI-60 consists of an automated scanning microscope, a high-quality digital camera, and a computer system with an acquisition and classification software from CellaVision named DI-60 Remote Review Software (version 7.0.2). The automated scanning microscope has two objectives (10× and 100×) with intermediate optics switching (1.0× and 0.5×), which can yield images with 5×, 10×, 50×, or 100× magnifications [16–19]. DI-60 supports three applications: PB, BF, and advanced red blood cell (RBC) [19]. In BF applications, DI-60 digitally scans the entire smear area of a cytocentrifuge-prepared slide at 10× or 50× magnifications, providing an overview area that allows the examiner to manually navigate and scan fields [9, 19, 20]. DI-60 can pre-classify BF cells into five types of leukocytes (neutrophils, lymphocytes, eosinophils, macrophages [including monocytes], and ‘other’ cells [basophils, lymphoma cells, atypical lymphocytes, blasts, and tumor cells]) and two types of non-leukocytes (smudge cells and artifacts) [19, 20]. As XN-350 and manual counting cannot count non-leukocytes such as smudge cells and artifacts, only leukocytes counted by DI-60 were included for comparison (**Table 2**). Similar to PB applications, the number of pre-classified cells can be set by the examiner in BF applications [18, 21]. In this study, we set DI-60 to pre-classify 250 cells because non-leukocytes were also pre-classified by DI-60. The pre-classified cells were verified and re-classified by a hematology expert.

**Table 2:** Body fluid cell categories differentially counted by DI-60, XN-350, and manual counting.

DI-60	XN-350	Manual counting
Neutrophils	Neutrophils	Neutrophils
Lymphocytes	Lymphocytes	Lymphocytes
Eosinophils	Eosinophils	Eosinophils
Macrophages including monocytes	Monocytes	Macrophages including monocytes
‘Other’ cells^†^	High-fluorescence body fluid cells^‡^	Basophils
Smudge cells		
Artifacts		

^†^‘Other’ cells include basophils, lymphoma cells, atypical lymphocytes, blasts, and tumor cells.

^‡^High-fluorescence body fluid cells include macrophages, mesothelial cells, and tumor cells.

XN-350 is one of the XN-L series instruments that can determine WBC counts and differentials using fluorescence flow cytometry and RBC counts using a direct current impedance method with hydrodynamic focusing for whole blood and BF [5, 6, 15, 22]. In the XN-BF mode, XN-350 aspirates 70 μL of BF for analysis and enumerates total nucleated cells (TNCs) including high-fluorescence BF cells (HF-BFs) and WBCs [22]. HF-BFs outside the WBC differential fluorescence scattergram include macrophages, mesothelial cells, and tumor cells [6]. WBC differential counts include neutrophils, lymphocytes, monocytes, and eosinophils [15]. As XN-350 cannot differentiate between macrophages, mesothelial cells, and tumor cells (HF-BFs) and classifies monocytes separately from macrophages, only neutrophils, lymphocytes, and eosinophils counted by XN-350 were included for comparison. DI-60 and XN-350 were performed according to the manufacturer’s instructions.

Manual differential counting was performed according to the CLSI H56-A guidelines [4]. Two hematology experts each counted at least 100 cells on each cytocentrifuge-prepared slide at 400× magnification, and the average values were obtained for evaluation. Discrepant data between the two experts were arbitrated by a third expert. In cases where the number of cells was sufficient, 200 cells were counted if possible. In cases where the number of cells was insufficient (< 100 cells), the number of cells counted and the percentage of each cell type subsequently calculated were recorded. In manual counting, BF cells were classified into neutrophils, lymphocytes, eosinophils, basophils, and macrophages (including monocytes) according to a laboratory protocol based on the CLSI H56-A and H20-A2 guidelines [4, 23].

### Statistical analysis

Data are expressed as the median (IQR) or number (percentage). The performance of DI-60 pre-classification based on verification using 213 samples was evaluated based on sensitivity, specificity, positive predictive value (PPV), negative predictive value, and accuracy with their 95% confidence interval (CI). The agreement between DI-60 pre-classification and verification was evaluated using Cohen’s kappa (κ) with the 95% CI, which was interpreted as follows: ≤ 0.20, none; 0.21–0.39, minimal; 0.40–0.59, weak; 0.60–0.79, moderate; 0.80–0.90, strong; > 0.90, almost perfect [24]. In addition, the performance and agreement of DI-60 were evaluated separately for CSF, pleural fluid, and ascites samples.

For the total samples, DI-60 pre-classification and verification were compared with BF cell differential counts obtained by XN-350 and/or manual counting. Wilcoxon test for paired samples, Bland-Altman plot analysis, and Passing-Bablok regression analysis were used for comparison. Pearson’s correlation coefficient (r) with the 95% CI was obtained and interpreted as follows: < 0.30, negligible; 0.30–0.50, low; 0.50–0.70, moderate; 0.70–0.90, high; 0.90–1.00, very high [25]. Statistical analyses were performed using MedCalc statistical software (version 20.109; MedCalc Software, Ostend, Belgium) and Microsoft Excel software (version 2016; Microsoft Corporation, Redmond, WA, USA). Statistical results were regarded as significant if the two-sided *P* value was less than 0.05.

### Ethics statement

This *in vitro* comparative study was conducted according to the Declaration of Helsinki. The Institutional Review Board (IRB) of CAUH approved the study protocol (IRB No. 2207-008-513). As this study was conducted using residual BF samples and cytospin slides after the requested test was performed, informed consent was waived according to the IRB policy. The data were analyzed anonymously.

## Results

The overall sensitivity of DI-60 pre-classification based on verification was high for neutrophils, lymphocytes, and macrophages (range, 83.1–99.4%) and relatively low for eosinophils and ‘other’ cells (69.4% and 33.7%, respectively) (**Table 3**). The overall specificity and overall accuracy were high for all cell types (range, 95.3–99.7% and 94.3–99.3%, respectively). The performance was similar between the total samples and CSF (n = 47), pleural fluid (n = 80), and ascites (n = 86) samples; however, the sensitivity for ‘other’ cells was high at 93.3% when using CSF samples. The agreement between DI-60 pre-classification and verification was strong (κ = 0.89) when using total samples (**Table 4**). The results using CSF, pleural fluid, and ascites samples were similar with κ values of 0.88 (95% CI, 0.88–0.89), 0.86 (95% CI, 0.85–0.87), and 0.90 (95% CI, 0.90–0.91), respectively. Depending on the cell type, the agreement was weak for eosinophils (κ = 0.57) and none for ‘other’ cells (κ = 0.11). DI-60 counted and pre-classified 44,060 cells, of which 3,394 cells (7.7%) were verified to be misclassified. Cells pre-classified as ‘other’ and eosinophils were the most misclassified cells (92.5% and 51.4%, respectively). Among cells pre-classified as ‘other’, 85.2% and 5.3% of cells were verified as lymphocytes and neutrophils, respectively. Among cells pre-classified as eosinophils, 49.1% of cells were verified as neutrophils.

**Table 3:** Performance of DI-60 pre-classification based on verification.

Cell type	Number of cells	Sensitivity, % (95% CI)	Specificity, % (95% CI)	Positive predictive value, % (95% CI)	Negative predictive value, % (95% CI)	Accuracy, % (95% CI)
**Total samples (n = 213)**						
Neutrophils	11,075	95.9 (95.5–96.2)	99.7 (99.6–99.7)	99.0 (98.9–99.2)	98.6 (98.4–98.7)	98.7 (98.6–98.8)
Lymphocytes	11,926	83.1 (82.4–83.7)	99.6 (99.6–99.7)	99.1 (98.9–99.2)	92.5 (92.2–92.8)	94.3 (94.1–94.5)
Eosinophils	397	69.4 (63.6–74.8)	99.5 (99.5–99.6)	48.6 (44.7–52.6)	99.8 (99.8–99.8)	99.3 (99.3–99.4)
Macrophages	18,443	99.4 (99.3–99.5)	96.5 (96.3–96.7)	95.0 (94.7–95.3)	99.6 (99.5–99.7)	97.7 (97.5–97.8)
‘Other’ cells	2,219	33.7 (29.6–38.1)	95.3 (95.1–95.5)	7.5 (6.6–8.4)	99.2 (99.2–99.3)	94.6 (94.4–94.8)
**CSF (n = 47)**						
Neutrophils	2,693	94.7 (93.8–95.5)	99.6 (99.4–99.8)	99.4 (99.0–99.6)	96.7 (96.2–97.2)	97.7 (97.3–98.0)
Lymphocytes	2,464	86.1 (84.8–87.3)	99.7 (99.5–99.9)	99.5 (99.1–99.7)	91.8 (91.1–92.5)	94.4 (93.9–94.9)
Eosinophils	124	69.0 (55.5–80.5)	98.8 (98.6–99.1)	32.3 (26.6–38.5)	99.7 (99.6–99.8)	98.6 (98.3–98.9)
Macrophages	1,629	99.6 (99.2–99.9)	98.6 (98.2–98.9)	95.0 (93.8–95.9)	99.9 (99.8–100.0)	98.8 (98.5–99.0)
‘Other’ cells	391	93.3 (68.1–99.8)	94.8 (94.3–95.3)	3.6 (3.0–4.2)	100.0 (99.9–100.0)	94.8 (94.3–95.3)
**Pleural fluid (n = 80)**						
Neutrophils	4,981	95.5 (94.9–96.1)	99.5 (99.4–99.6)	98.9 (98.5–99.1)	98.1 (97.8–98.3)	98.3 (98.1–98.5)
Lymphocytes	4,756	80.0 (78.9–81.0)	99.4 (99.2–99.5)	98.5 (98.1–98.8)	90.5 (90.1–91.0)	92.7 (92.3–93.1)
Eosinophils	219	71.3 (64.2–77.6)	99.5 (99.4–99.6)	61.2 (55.6–66.5)	99.7 (99.6–99.7)	99.2 (99.0–99.3)
Macrophages	6,081	99.0 (98.7–99.2)	96.2 (95.8–96.5)	92.8 (92.1–93.4)	99.5 (99.3–99.6)	97.1 (96.8–97.3)
‘Other’ cells	1,091	33.3 (27.3–39.8)	94.0 (93.6–94.4)	7.1 (5.9–8.4)	99.0 (98.9–99.1)	93.2 (92.8–93.6)
**Ascites (n = 86)**						
Neutrophils	3,401	97.3 (96.8–97.9)	99.8 (99.8–99.9)	99.2 (98.8–99.5)	99.4 (99.3–99.5)	99.4 (99.3–99.5)
Lymphocytes	4,706	84.8 (83.8–85.7)	99.8 (99.7–99.9)	99.5 (99.2–99.6)	94.4 (94.0–94.7)	95.6 (95.3–95.9)
Eosinophils	54	59.4 (40.6–76.3)	99.8 (99.8–99.9)	35.2 (25.9–45.7)	99.9 (99.9–100.0)	99.8 (99.7–99.8)
Macrophages	10,733	99.7 (99.5–99.8)	95.6 (95.2–96.0)	96.2 (95.9–96.6)	99.6 (99.5–99.7)	97.8 (97.5–98.0)
‘Other’ cells	737	30.5 (24.8–36.7)	96.6 (96.3–96.8)	10.2 (8.5–12.2)	99.1 (99.0–99.2)	95.8 (95.5–96.0)

Abbreviations: CI, confidence interval; CSF, cerebrospinal fluid.

**Table 4:** Agreement between DI-60 pre-classification and verification for cells in body fluid.

Total samples (n = 213), κ = 0.89 (95% CI, 0.88–0.89)						
Pre-classification	Verification					κ (95% CI)
	Neutrophils	Lymphocytes	Eosinophils	Macrophages	‘Other’ cells	
Neutrophils	10,974	0	54	47	0	0.97 (0.96–0.97)
Lymphocytes	77	11,816	4	26	3	0.86 (0.86–0.87)
Eosinophils	195	3	193	4	2	0.57 (0.52–0.61)
Macrophages	86	515	4	17,517	321	0.95 (0.95–0.96)
‘Other’ cells	117	1,891	23	22	166	0.11 (0.09–0.12)

Abbreviations: CI, confidence interval; CSF, cerebrospinal fluid.

DI-60 pre-classification showed significant differences from DI-60 verification for all cell types except neutrophils (*P* = 0.24). Both DI-60 pre-classification and verification showed significant differences from XN-350 for all cell types (*P* < 0.01, respectively) except macrophages and ‘other’ cells, which were not available for comparison as the cell types classified by DI-60 and XN-350 are different. Both DI-60 pre-classification and verification showed significant differences from manual counting for lymphocytes and macrophages (*P* < 0.01, respectively). The absolute mean differences between DI-60 pre-classification and XN-350 ranged from 0.78 to 3.48; after verification, differences between DI-60 and XN-350 ranged from 0.26 to 11.05 (**Table 5**).

**Table 5:** Comparison of body fluid cell differential counts obtained by DI-60, XN-350, and manual counting (n = 213).

Cell type	DI-60, % (median, IQR)		XN-350^†^, % (median, IQR)	Manual counting, % (median, IQR)	Mean difference, % (95% CI)				
	Pre-classification	Verification			Pre-classification vs. verification	XN-350^†^ vs. DI-60		Manual counting vs. DI-60	
						Pre-classification	Verification	Pre-classification	Verification
Neutrophils	8.1 (2.5–37.1)	8.5 (2.3–38.5)	18.5 (8.1–54.5)	8.5 (2.1–41.9)	1.56 (0.89 to 2.23)	3.18 (1.50 to 4.87)	-0.96 (-3.22 to 1.29)	-0.72 (-1.48 to 0.05)	0.14 (-0.37 to 0.64)
Lymphocytes	22.8 (11.5–39.5)	28.0 (11.6–50.9)	79.7 (39.8–91.6)	36.8 (14.7–57.2)	5.23 (4.04 to 6.41)	-3.48 (-5.31 to -1.65)	-11.05 (-13.80 to -8.30)	-7.78 (-9.03 to -6.53)	-4.76 (-5.74 to -3.78)
Eosinophils	0.4 (0.0–0.9)	0.0 (0.0–0.4)	0.0 (0.0–1.1)	0.0 (0.0–0.8)	-0.02 (-0.39 to 0.36)	0.78 (0.34 to 1.21)	0.26 (-0.13 to 0.64)	0.09 (-0.25 to 0.42)	0.01 (-0.12 to 0.13)
Macrophages	39.2 (16.7–61.5)	35.4 (13.5–59.9)	NA	29.6 (11.5–53.8)	-2.36 (-2.99 to -1.73)	NA	NA	8.39 (7.19 to 9.59)	4.52 (3.50 to 5.54)
‘Other’ cells	4.5 (2.0–7.7)	0.4 (0.0–2.2)	NA	NA	-4.41 (-5.25 to -3.57)	NA	NA	NA	NA

^†^Sysmex XN-350 failed to count cells in three cerebrospinal fluid samples. The results of XN-350 are for 210 samples.

Abbreviations: CI, confidence interval; IQR, interquartile range; NA, not available.

**Fig 1:**
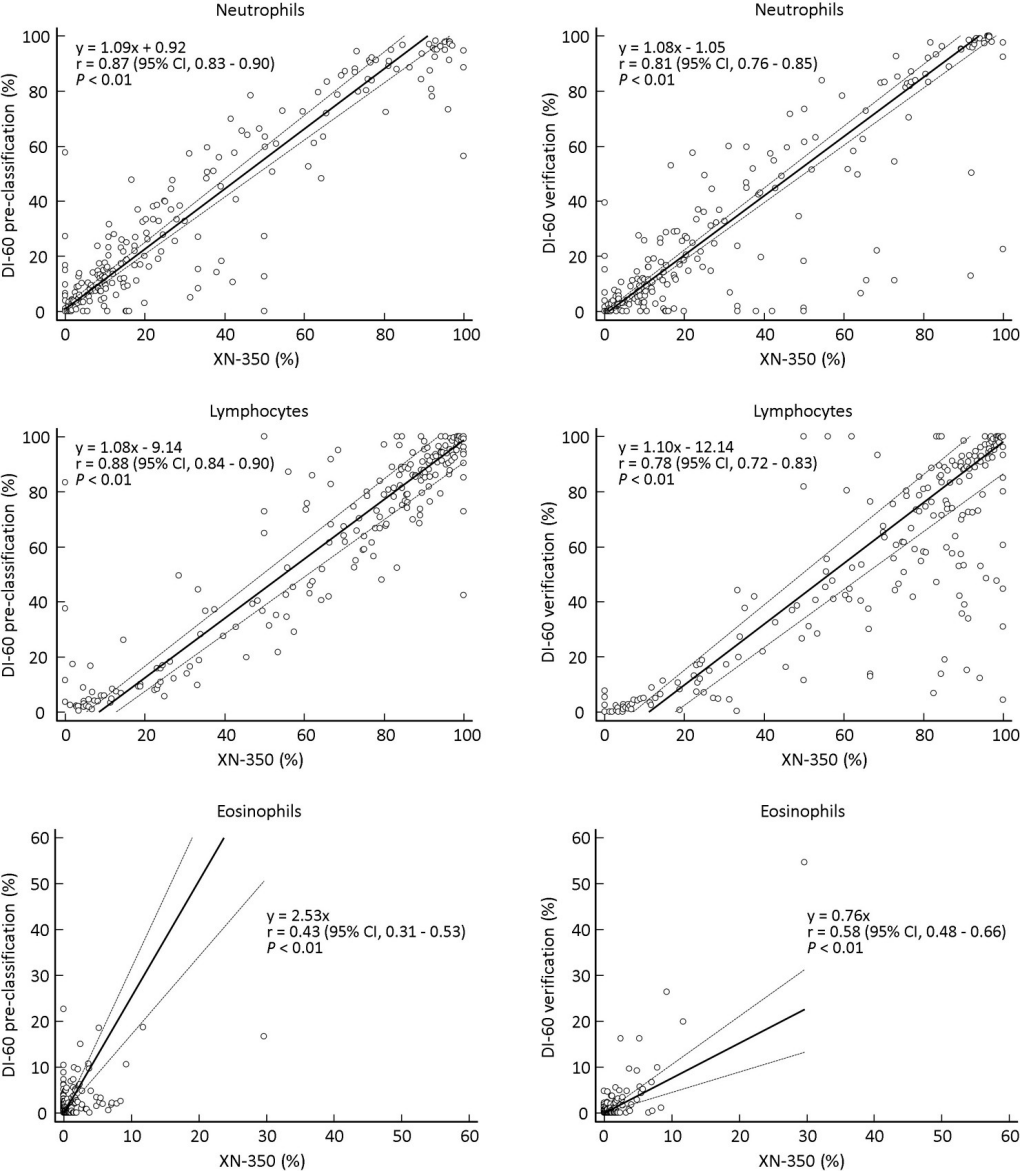
Comparison between DI-60 and XN-350 (n = 213). Solid line, Passing-Bablok regression; dashed line, 95% confidence interval line.

**Fig 2:**
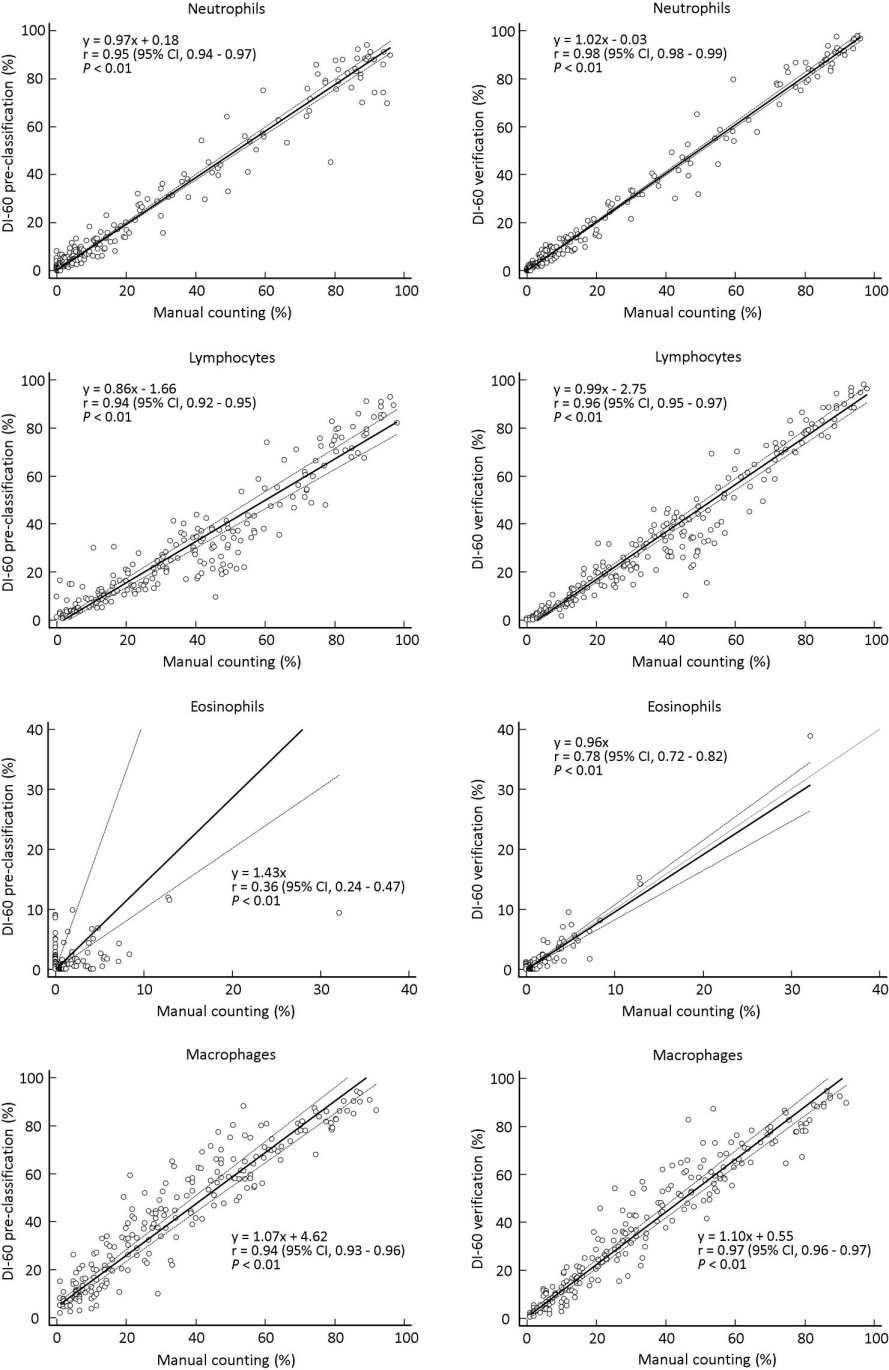
Comparison between DI-60 and manual counting (n = 213). Solid line, Passing-Bablok regression; dashed line, 95% confidence interval line.

The absolute mean differences between DI-60 pre-classification and manual counting ranged from 0.09 to 8.39; after verification, differences between DI-60 and manual counting ranged from 0.01 to 4.76. DI-60 pre-classification and XN-350 showed a high correlation for neutrophils (r = 0.87) and lymphocytes (r = 0.88) and a low correlation for eosinophils (r = 0.43) (**Fig 1**). After verification, the correlation between DI-60 and XN-350 was lower for neutrophils and lymphocytes but was improved for eosinophils. DI-60 pre-classification and manual counting showed a very high correlation for neutrophils (r = 0.95), lymphocytes (r = 0.94), and macrophages (r = 0.94) and a low correlation for eosinophils (r = 0.36) (**Fig 2**). After verification, the correlation between DI-60 and manual counting was improved for all cell types (r = 0.78 to 0.98).

## Discussion

BF samples, especially CSF, may be characterized by the instability of cellular constituents due to their chemical composition [4, 7]. Therefore, BF samples should be examined within a short period of time to minimize the effect of variables related to sample storage [4, 7, 13]. The use of automated analyzers including automated hematology analyzers and DM analyzers may be an efficient option in terms of time and cost for the cell differential counting of BF and PB, which are known to have excellent reproducibility and accuracy [5, 7, 11, 15]. Previous studies have compared automated hematology analyzers or DM analyzers with only manual counting using BF samples [1–3, 5–11, 14, 15]. In this study, we comprehensively evaluated the analytical performance of DI-60 for BF cell differential counts, including sensitivity, specificity, accuracy, and agreement. We also compared BF cell differential counts obtained by DI-60 with those obtained by XN-350 and manual counting.

DI-60 pre-classification of BF cells based on verification showed high accuracy when using not only total samples but also CSF, pleural fluid, and ascites samples (**Table 3**). DI-60 showed high sensitivity for ‘other’ cells in CSF samples, which was low when using pleural fluid and ascites samples. This result indicated the low number of misclassified ‘other’ cells in CSF samples. Although there are few cells in the ‘other’ cells category of CSF, DI-60 seems to be able to sensitively detect them. Regardless of the BF sample type, DI-60 demonstrated low PPVs and weak/no agreement for eosinophils and ‘other’ cells. A large number of the cells pre-classified as eosinophils and ‘other’ cells by DI-60 were misclassified (**Table 4**). In a previous study evaluating DM96, another DM analyzer for BF cell differential counts, the agreement was relatively low for eosinophils and ‘other’ cells (55.8% and 28.9%, respectively), which is consistent with the results of our study [7]. The findings may be attributed to the low cell count of eosinophils and ‘other’ cells in BF and differences in the proportions of cells detected at different locations on the slide [7, 26–28]. A critical drawback of DM analyzers, including DI-60, is that they cannot track where the detected cells are located on the slide.

The absolute mean differences between DI-60 pre-classification and XN-350 were acceptable for all cell types (neutrophils, lymphocytes, and eosinophils); however, differences between DI-60 and XN-350 for lymphocytes were greater after verification. Differences between DI-60 and manual counting were decreased after verification for all cell types (neutrophils, lymphocytes, eosinophils, and macrophages) and were acceptable (**Table 5**). The correlation between DI-60 pre-classification and XN-350 was high for all cell types except for eosinophils; however, it was lower for neutrophils and lymphocytes after verification (**Fig 1**). On the other hand, the correlation between DI-60 pre-classification and manual counting was very high for all cell types except for eosinophils, which was improved after verification for all cell types (**Fig 2**). The findings are similar to those of a previous study comparing BF cell differential counts between DI-60 and manual counting using 34 CSF samples and 60 other BF samples [8]. The low r value for eosinophils indicates a lower accuracy for these cells compared with other cell types. Similarly, a low correlation between the DM analyzer and manual counting has been observed for basophils in PB samples, which can be explained by a low cell count [17, 18, 26–28]. Our findings suggest that verification of the results by DM analyzers and manual slide review are still required for BF and PB samples [12, 27]. In addition, the correlation between DI-60 and XN-350, which was lower after verification, implies that BF cell differential counts obtained by DI-60 could not replace those obtained by XN-350. DI-60 and XN-350 differ in sample handling and cell type. For DI-60, a cytocentrifugation step is added during sample handling, which can improve the correlation between DI-60 and manual counting. Given that BF cell differential counting using cytospin slides is the standard method for the morphological assessment of BF, the BF cell differential count results of XN-350 remain insufficient. This study included samples from patients diagnosed with infectious/inflammatory diseases. Under infectious/inflammatory conditions, the WBC could have alterations in size and cellular contents, which could significantly modify the results of XN-350 by affecting the forward scatter and side scatter parameters used for flow cytometry.

This study provides baseline data on the analytical performance of DI-60 for BF cell differential counts using CSF, pleural fluid, and ascites samples. However, there are several limitations in this study. First, the cytospin slides used in this study were stained only with Wright-Giemsa from RAL Diagnostics using SP-50. The performance of DM analyzers may vary depending on the staining method and/or slide maker [29, 30]. Therefore, the performance should be further compared between different slide-staining/making methods. In addition, the performance of DM analyzers is highly dependent on the quality of the slide and staining [12, 31]. Clinical laboratories need to perform regular internal and external quality controls of slides and DM analyzers, even for BF samples [12, 13]. Second, we compared different methods, including different analytical purposes and sample preparation procedures. DI-60 is an automated image analysis system for morphology assessment, and Romanowsky-stained slides of cytocentrifuged BF are used for analysis [4, 12]. On the other hand, XN-350 is an automated cell counter for quantitative assessment and requires no special sample preparation [4, 13, 22]. Sample preparation may result in cell deformity or cell lysis, which may affect the analysis [8]. Therefore, it is important to consider that several factors may affect the comparison between different methods. Third, we did not evaluate the performance of DI-60 for the detection of tumor cells in BF samples. Only one pleural fluid sample and two ascites samples contained tumor cells. Although the number of samples with tumor cells was small, DI-60 identified tumor cells in all three samples and pre-classified them into the ‘other’ cells category. However, not all tumor cells were pre-classified into the ‘other’ cells category, and a considerable number of cells were pre-classified into artifacts category. This outcome may be explained by the formation of clusters from tumor cells, and the detection of such clusters is still difficult [4, 9].

In conclusion, this is the first study to evaluate the performance of DI-60 compared with XN-350 and manual counting for BF cell differential counts. DI-60 showed reliable analytical performance with improvement after verification when using CSF, pleural fluid, and ascites samples. For BF cell differential counts, DI-60 may be a better option than XN-350 and could be used for screening purposes in understaffed laboratories. However, DI-60 cannot completely replace the gold standard method, i.e., manual counting. Verification and manual slide review are still required, especially for samples with large numbers of cells pre-classified into the eosinophils and ‘other’ cells categories. To improve the hematology workflow for BF cell differential counting, the DM analyzer needs to be optimized by taking into account the laboratory situation and unmet needs, and the clinical laboratory needs to establish criteria for verification and manual slide review.
